# Galacto-oligosaccharides alleviate experimental lactose intolerance associated with gut microbiota in mice

**DOI:** 10.3389/fmicb.2025.1530156

**Published:** 2025-03-25

**Authors:** Qianxi Li, Xinlei Wang, Siyu Guo, Tongtong Wang, Heng Cao, Yunhe Cao, Bing Dong

**Affiliations:** State Key Laboratory of Animal Nutrition and Feeding, College of Animal Science and Technology, China Agricultural University, Beijing, China

**Keywords:** galacto-oligosaccharides, lactose intolerance, gut microbiota, mice, lactase

## Abstract

**Introduction:**

Galacto-oligosaccharides (GOS) are beneficial for alleviating lactose intolerance (LI). GOS have the ability to modify the composition of the intestinal microbiota. The development of intestinal diseases could be influenced by the composition of the gut microbiota. Nevertheless, it remains unclear whether gut microbiota exerts an effect when GOS alleviate LI, whether alterations in composition of the intestinal microbiota influence inflammatory response and lactose digestion.

**Methods:**

We first investigated the effects of GOS on mice with established lactose intolerance. Next, we demonstrated that prophylactic supplementation with GOS also conferred similar benefits.

**Results:**

The results showed that GOS enhanced anti-inflammatory, antioxidant, and gut barrier function. We observed that GOS mediated a change in the gut microbiome by increasing the abundance of *Lactobacillus*. GOS pre-supplementation reduced incident LI, enhanced anti-inflammatory, antioxidant, and gut barrier function, and markedly altered the gut microbiome by significantly enriching *Bifidobacterium*. Collectively, the alleviation of LI by GOS suggests an intimate involvement of probiotics.

**Discussion:**

This study demonstrates that GOS ameliorated LI in a gut microbiota-dependent manner. Our findings provide novel evidence that GOS substitute for lactase and serve as a potential modulator of the gut microbiota for the prevention of LI.

## 1 Introduction

Lactose intolerance (LI) is a global issue in public health management. Approximately two-thirds of the population have LI all over the world (Toca et al., 2022). The clinical manifestations of LI encompass abdominal distension, abdominal pain, nausea, increased intestinal peristalsis, and diarrhea. Accumulation of undigested lactose in the intestinal lumen leads to an elevated osmotic pressure which cause hyperosmotic diarrhea. Simultaneously, the fermentation of lactose by intestinal microbiota generates volatile fatty acids and gases such as hydrogen, methane, and carbon dioxide (Vandenplas, 2015). Currently, the primary therapeutic strategies for LI involve restricting intake of lactose-containing dairy products (milk, soft cheese, and ice cream) or using lactase supplements concurrent with dairy consumption. Blind avoidance of dairy products results in a significant decrease in calcium intake and increased risk of chronic diseases such as low bone density, osteoporosis and their sequelae among many lactose intolerant patients (Obermayer-Pietsch et al., 2004; Ross et al., 2011). Interestingly, elevated milk intake among lactose intolerant individuals decreases risk of type 2 diabetes (Luo et al., 2024). Hence, it is of great significance to assist lactose intolerant patients in developing tolerance to lactose.

Intestinal tolerance to lactose could be established through adaptation of the intestinal microbiota (Szilagyi et al., 2010; Azcarate-Peril et al., 2021). This adaptive mechanism encompasses increasing abundance and diversity of lactose-metabolizing bacteria which induces production of β-galactosidase by microorganisms (Hertzler and Savaiano, 1996), and enhances utilization rate of hydrogen generated during fermentation (Hertzler et al., 1997). These adaptions enable rapid metabolism of lactose by the intestinal microbiota. Intestinal microbiota served as a crucial bridge between environmental factors and host health (Kamada et al., 2013). Galacto-oligosaccharides (GOS) can alter the composition of the intestinal microbiota, as a “Bifidus Factor,” by increasing abundances of *Bifidobacterium* (Andersen et al., 2011). Dysbiosis of the intestinal microbiota is a characteristic of intestinal diseases. One therapeutic strategy to correct dysbiosis is to increase the proportion of beneficial bacteria in the intestinal microbiota (Zhang et al., 2020).

Researched have evaluated whether GOS alleviates clinical symptoms of LI in humans, using the hydrogen breath test (HBT) and conducting regular follow-up exams. Savaiano et al. (2013) continuously supplemented LI patients with a GOS formulation for 35 days. Subsequently, the patients reintroduced dairy products into their daily diet and underwent an additional 30-day follow-up. Chey et al. (2020) also conducted similar researches. These studies revealed patients exhibited a tendency for improvement in both lactose digestion and lactose intolerance symptoms. However, the specific mechanism remains unclear. Studies of GOS supplementation in human are useful but have significant flaws. The inconsistent diet of lactose intolerant patients, the strong subjectivity of patient reports, and the small sample size are significant limitations of human studies. Therefore, it is necessary to establish animal models to further explore the underlying mechanisms.

Lactase activity of C57BL/6 mice is extremely high before weaning, but drops sharply after weaning. Lactase activity of these mice 8 weeks postweaning is very low, and similar to that of adult humans (Detel et al., 2008). C57BL/6 mice after weaning can serve as an ideal animal model for the study of lactose intolerance.

In this study, to explore the potential alleviative effect of GOS in treatment or prevent of lactose intolerance, we investigated the effect of GOS in lactose induced LI mice and measured the indices related to intestinal inflammation, oxidative stress, and barrier dysfunction and the alteration of intestinal microbiota. The underlying mechanisms of GOS were also studied.

## 2 Materials and methods

### 2.1 Animals

Eight-week-old male mice (C57BL/6N) status (SPF Biotechnology Co., Ltd., Beijing, China), with an initial body weight of 21.56 ± 0.13 g were used in this study. Feed (AIN-93) required for the mice during the experiment was sourced from Xiaoshu Youtai Biotechnology Co., Ltd (Beijing, China). Six animals were housed in each cage and kept in the standard specific pathogen-free (SPF) facilities of China Agricultural University. The light/dark cycle in the experimental rooms was 12 h of light and 12 h of darkness daily, with a temperature of 22°C and a humidity of 50%. The experimental protocol was approved by the Animal Experiment Ethics Committee of the Experimental Animal Center of China Agricultural University, with the approval number AW91603202-1-1. The experimental process adhered to the “Authors’ Guide to Consensus on Animal Ethics and Welfare” of the International Association of Veterinary Editors and local and national regulations. Mice were dosed with lactose (molecular weight 342, purity ≥ 98%, Macklin Biochemical Technology Co., Ltd, Shanghai, China). GOS (0.5 g/d, purity ≥ 98%, Macklin Biochemical Technology Co., Ltd, Shanghai, China) was administered to mice by oral gavage. The oligomers of GOS was 3 to 5. Lactase (Cangzhou SUNSON Enzyme Bioctechnology Co., Ltd, Hebei, China).

### 2.2 Treatments and sample collection

To ascertain the success of lactose intolerance modeling, Experiment 1 was conducted, a total of 16 mice (*n* = 8/group). After one-week of acclimation, mice were divided randomly into two treatment groups, with 8 mice in each group. To build the LI model, mice, fed a normal diet of AIN-93 (Supplementary Table 1), were gavaged with 200 μL saline containing lactose (50 mg/20 g BW) with the intragastric needle daily. Mice in the experimental group (lac) were gavaged orally with lactose for 7 days to build up lactose intolerance, while mice in the control group (con) were gavaged with an equal volume of saline. After 7 days, mice were sacrificed, and their histological changes, changes in concentration of blood inflammatory factors level. The intestinal tissues of the ileum and jejunum were subjected to histological examination by Hematoxylin and Eosin (H&E) staining. The diarrhea condition was measured after lactose challenging, mice were fasted for 12 h in advance, and then were allowed to access food and water. The number of defecations and the total fecal mass were recorded within 6 h after lactose administration (Li et al., 2012).

**FIGURE 1:**
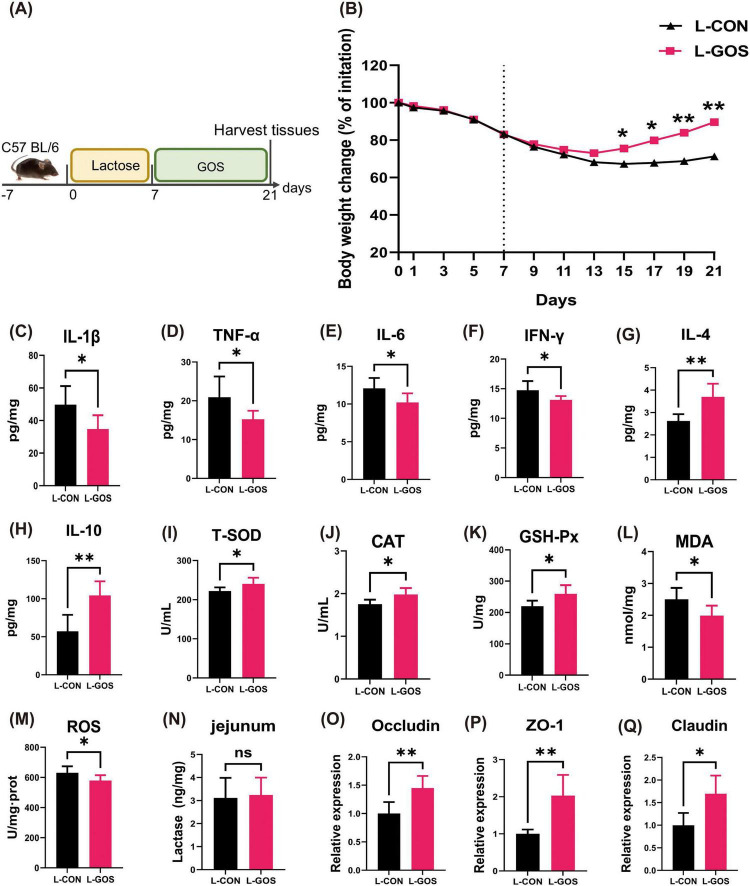
GOS alleviated lactose-induced experimental LI. **(A)** Diagram illustrating the mouse model of LI employed in this study. **(B)** Oral normal saline and GOS treatments are indicated. Daily body weight changes throughout the entire duration of the study. **(C–H)** Concentrations of four representative pro-inflammatory cytokines, IL-1β, TNF-α, IL-6, IFN-γ, and anti-inflammatory cytokines, IL-4, IL-10 in the ileum. **(I–M)** GOS attenuated oxidative stress and jejunal damage. Concentrations of T-SOD, CAT, GSH-Px, MDA and ROS in the ileum from each group. **(N)** Concentrations of β-galactosidase in jejunum. **(O–Q)** Relative expression jejunal level of Occludin, ZO-1, Claudin mRNA in mice. Data were presented as means ± SEM (*n* = 8 per group). Statistical significance was determined using one-way ANOVA, followed by Tukey test. *P ≤ 0.05, **P ≤ 0.01.

To investigate if GOS could mitigate lactose intolerance, Experiment 2 was conducted with a total of 16 mice (*n* = 8/group). After one week of acclimatization, male mice were assigned randomly to one of two treatment groups, with 8 mice in each group (Figure 1A). Mice were gavaged orally with lactose for 7 days to build up lactose intolerance, and then daily gavaged with saline (200 μL) or the same volume of saline containing 0.5 g of GOS for 14 days. The treatment groups were as follows: (1) L-CON group: gavaged with lactose for 7 days and followed by saline for 14 days; (2) L-GOS group: gavaged with lactose for 7 days and followed by GOS for 14 days.

Throughout the entire research period, body weight of mice was measured daily. On the 21st day, mice were sacrificed, and cecal chyme samples from all mice were collected aseptically and stored at −80°C for future analysis. The middle segments of the jejunum (2 cm) and ileum (2 cm) were rinsed with physiological saline and rapidly frozen in liquid nitrogen for measurement of inflammatory cytokines, antioxidant indices, barrier injury and lactase.

To investigate the potential preventive effect of GOS in LI mice, Experiment 3 was conducted with a total of 36 mice (*n* = 6/group). After one week of acclimation, mice were divided randomly into six groups, with 6 mice in each group (Figure 3A). For three weeks, mice were gavaged orally with saline (200 μL) or the same volume of saline containing GOS daily. Subsequently, mice were gavaged orally for 5 days with saline (200 μL) or the same volume of saline containing lactose daily to induce lactose intolerance. The treatment groups were as follows: (1) CON group: daily gavage with saline for three weeks,; (2) GOS group: daily gavage with saline containing 0.5 g of GOS for three weeks; (3) Gal group: daily gavage with saline containing lactase for three weeks; (4) CON-L group: daily gavage with saline for three weeks, followed by five consecutive days of lactose gavage; (5) GOS-L group: daily gavage with saline containing 0.5 g of GOS for three weeks, followed by five consecutive days of lactose gavage; (6) Gal-L group: daily gavage with saline containing lactose for three weeks, and finally five days of lactose gavage. Thirty minutes before the daily gavage of lactose, lactase was gavaged first. CON, GOS and Gal groups were gavaged with an equal volume of saline at 21–26 days. From day 21 to day 26, body weight was measured daily. All mice were euthanized under anesthesia on the 26th day. Cecal chyme samples from all mice were collected aseptically and stored at −80°C for future analysis. The middle segments of the jejunum and ileum were rinsed with saline and rapidly frozen in liquid nitrogen for measurement of inflammatory cytokines, antioxidant indexes, barrier injury and lactase.

### 2.3 Quantification of inflammatory cytokines, antioxidant indexes in the ileum and barrier injury in the jejunum

A part of the frozen ileum samples was homogenized with RIPA lysis buffer (Solarbio, Beijing, China) to extract total protein. The homogenate was centrifuged at 12,000 × *g* at 4°C for 15 min. Protein content was quantified using a bicinchoninic acid (BCA) protein assay kit (Solarbio, Beijing, China) in accordance with the manufacturer’s instructions. Concentrations of pro-inflammatory cytokines such as IL-1β, IL-6, TNF-α, and IFN-γ, and anti-inflammatory cytokines such as IL-4 and IL-10 in ileal tissue were measured using an enzyme-linked immunosorbent assay (ELISA) (R&D Systems, Minneapolis, MN, USA) as per the manufacturer’s recommendations. Concentrations of representative redox enzymes such as total superoxide dismutase (T-SOD), catalase (CAT), glutathione peroxidase (GSH-Px), and concentrations of malondialdehyde (MDA) were quantified using commercial kits (Nanjing Jiancheng Bioengineering Institute, Nanjing, China), and the concentration of reactive oxygen species (ROS) was quantified using the probe method. To assess the effect of GOS on the integrity of the intestinal barrier, mRNA expression levels of tight junction proteins Occludin, ZO-1, and Claudin in the jejunum of mice were measured.

### 2.4 DNA extraction and 16S rRNA gene sequencing

Total microbial genomic DNA in each sample of cecal chyme was extracted using the E.Z.N.A.^®^ Soil DNA Kit (Omega Bio-Tek, USA). Concentration and integrity of the DNA were evaluated using Agarose 75510019 (Thermo Fisher Scientific, USA) and 1.5% agarose gel electrophoresis, respectively. Subsequently, diluted DNA (1.0 ng/mL) was used to amplify the V3-V4 hypervariable region of the 16S rRNA gene with primers (forward primer 338F: ACTCCTACGGGAGGCAGCAG; reverse primer 806R: GGACTACHVGGGTWTCTAAT). The PCR products were subjected to 2.0% agarose gel electrophoresis, recovered, and purified using the GeneJET Gel Extraction Kit (Thermo Fisher Scientific, USA). The PCR products were quantified using Qubit 4.0 (Thermo Fisher Scientific, USA). According to sequencing requirements for each sample, samples were mixed in the corresponding proportions. The library was constructed using the NEXTFLEX Rapid DNA-Seq Kit and sequenced on the Illumina Miseq PE300 platform (Shanghai Majorbio Biomedical Technology Co., Ltd., Shanghai, China). The microbial sequencing results of Experiments 2 and 3 were deposit to NCBI (BioProject ID: PRJNA1209674 and PRJNA1209660).

### 2.5 Statistical analysis

All data are presented as the mean ± SEM and analyzed using the GraphPad Prism 9.5 program (GraphPad Software, San Diego, CA, USA). Data from two groups were analyzed using the *t*-test, and data from more than two groups were analyzed using one-way ANOVA followed by Tukey’s multiple comparison test. An adjusted *P* < 0.05 was considered statistically significant.

All data analyses were performed on the Majorbio Cloud Platform^1^ as follows: The Alpha diversity metrics such as Chao, Sobs and Shannon index were calculated using the mothur (Schloss et al., 2009) software,^2^ and the inter-group differences in Alpha diversity were analyzed using the Wilcoxon rank sum test. The PCoA analysis (principal coordinate analysis) based on the Bray-Curtis distance algorithm was used to examine similarity of the microbial community structure among samples, and the PERMANOVA non-parametric test was combined to analyze whether the differences in the microbial community structure among sample groups were significant. Based on a Spearman correlation of |r| > 0.6 and *p* < 0.05, species were selected for the correlation network diagram analysis (Barberán et al., 2012).

## 3 Results

### 3.1 Successful induction of lactose intolerance model

Obvious changes in intestinal morphology could be observed (Supplementary Figures 1a–d). The tips of the intestinal villi were incomplete, and a large number of white blood cells were aggregated. The levels of intestinal pro-inflammatory cytokines IL-1β and TNF-α significantly increased (Supplementary Figures 1e,f). On the 7th day, 6 h after the lactose challenge, no significant difference in fecal frequency was found between the two groups. Compared with the con group mice, the total fecal weight significantly increased in the lac group (Supplementary Figure 1g). The picture (Supplementary Figure 1h) showed the LI mice intestinal morphology on day 7 of lactose challenge.

### 3.2 GOS promoted recovery after lactose intolerance in mice

#### 3.2.1 GOS alleviated LI-induced intestinal inflammation

Experiment 1 (Figure 1A). Mice fed with lactose for seven days lost body weight through day 15 of the experiment (Figure 1B). Even after cessation of lactose feeding, the body weight still exhibited a downward trend. Mice treated with GOS demonstrated reduced weight loss, an earlier inflection point for weight recovery, and a faster recovery tendency.

To assess the impact of GOS on the occurrence of inflammation, levels of inflammatory factors in the ileum of mice were measured (Figures 1C–H). GOS significantly decreased the levels of IL-1β, TNF-α, IL-6, and IFN-γ in lactose-intolerant mice, while significantly elevated levels of IL-4 and IL-10. In comparison with the L-CON, L-GOS significantly mitigated the level of intestinal inflammation following the occurrence of lactose intolerance, mainly characterized by a marked decline in the content of intestinal pro-inflammatory factors and a significant increase in the content of anti-inflammatory factors.

#### 3.2.2 GOS alleviated LI-induced intestinal oxidative stress

After GOS supplementation in mice with lactose intolerance, the levels of T-SOD, CAT, and GSH-Px in the ileum of lactose intolerant mice were significantly higher than those in the control group (Figures 1I–M), while the levels of MDA and ROS were significantly lower than those in the control group. The results indicated that GOS intervention significantly mitigates the level of intestinal oxidative stress following the occurrence of lactose intolerance. This mitigation was manifested primarily by a decline in intestinal oxidative stress, an increase in antioxidant enzyme content, and an enhancement in antioxidant capacity.

#### 3.2.3 GOS ameliorated LI-induced intestinal barrier injury

Administration of GOS to LI mice had no statistically significant effect on lactase content of the jejunum (Figure 1N), suggesting that GOS did not affect the expression of intestinal lactase. Presumably, the beneficial role of GOS in alleviating lactose intolerance was not achieved through increasing activity of intestinal lactase.

After administered of GOS, mRNA expression levels of Occludin, ZO-1, and Claudin proteins in the jejunum of lactose intolerant mice (Figures 1O–Q) exhibited significant elevations, which suggests that GOS significantly alleviated intestinal barrier damage caused by the occurrence of lactose intolerance and promoted restoration of intestinal barrier integrity.

#### 3.2.4 GOS regulated the composition of the intestinal microbiota

**FIGURE 2:**
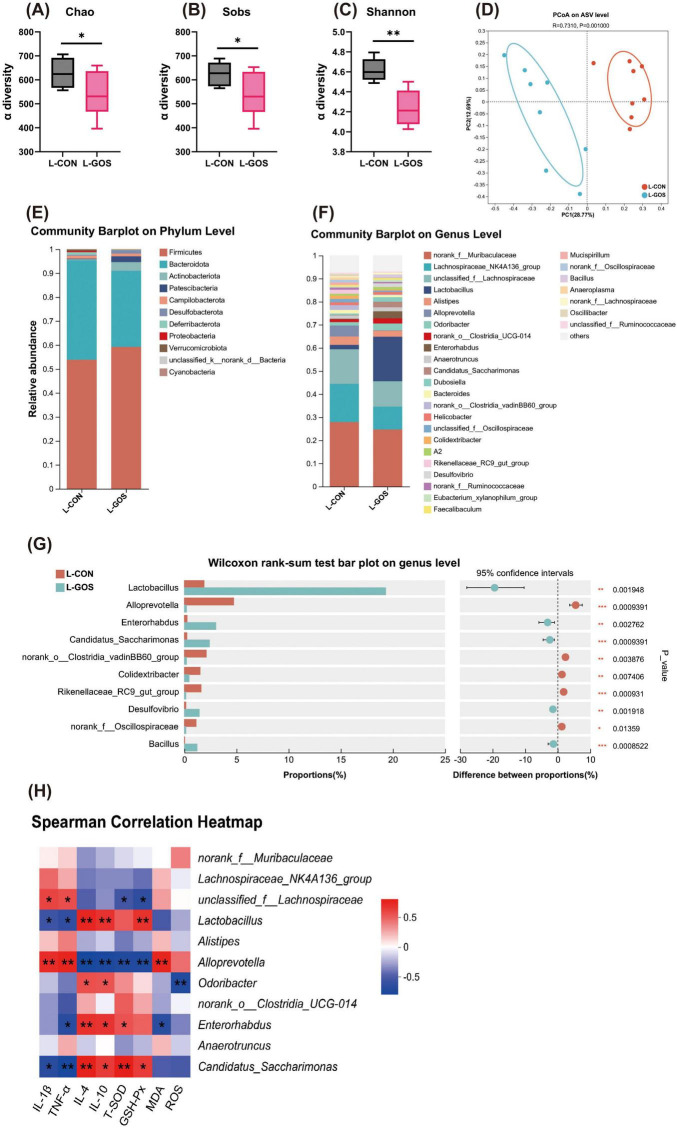
GOS regulated the composition and function of intestinal microbiota. **(A–C)** α-diversity upon GOS represented by the Chao, Sobs and Shannon index. **(D)** PCoA plots upon GOS assessed by PERMANOVA. **(E,F)** The relative abundance of colonic contents bacterial phyla and genera presented in 99.5% of the community upon GOS. **(G)** Analysis of differences in the microbial taxa shown by Wilcoxon rank-sum test on genus level upon GOS. **(H)** Spearman correlation between intestinal microbiota and anti-inflammatory or anti-oxidative parameters in two groups. The red color denotes a positive correlation, while blue color denotes a negative correlation. The intensity of the color is proportional to the strength of Spearman correlation. Statistical significance was determined using one-way ANOVA, followed by Tukey test. *P ≤ 0.05, **P ≤ 0.01, ***P ≤ 0.001.

The α-diversity of intestinal microbiota exhibited a significant decrease in LI mice that supplemented with GOS (Figures 2A–C). Principal co-ordinates analysis (PCoA) based on the Bray-Curtis distance revealed (Figure 2D) that intestinal microbiota populations of the L-CON and the L-GOS mice were significantly different (*R* = 0.731, *P* = 0.001). This observation indicates that GOS significantly affected the intestinal microbiota populations of lactose-intolerant mice.

At the phylum level of microbial composition, Bacteroidetes and Firmicutes are the dominant phyla in the cecal microbiota (Figure 2E). Administration of GOS altered the microbial composition, manifested as an increased proportion of Firmicutes, a decreased proportion of Bacteroidetes, and a significantly increased proportion of Actinobacteria. At the genus level, administration of GOS increased *Lactobacillus* (Figure 2F), and decreased *Lachnospiraceae*, and *Alloprevotella*. *Lactobacillus* belongs to the Firmicutes phylum, so the increased abundance of *Lactobacillus* corroborates the increased proportion of Firmicutes. Supplementation of LI mice with GOS, significantly increased the proportion of beneficial bacteria such as *Lactobacillus* and *Enterorhabdus*, and significantly reduced the proportion of *Alloprevotella* and *Colidextribacter* (Figure 2G).

The Spearmen correlation analysis (Figure 2H) indicated that *Lactobacillus* was significantly negatively correlated with IL-1β and TNF-α, and significantly positively correlated with IL-4 and IL-10. Furthermore, *Lactobacillus* was significantly positively correlated with GSH-Px, tended to be correlated positively with T-SOD and tended to be correlated negatively with MDA and ROS. *Enterorhabdus* also exhibited correlations similar to *Lactobacillus*. Correlations among *Alloprevotella* and inflammatory and antioxidant parameters were completely opposite to *Lactobacillus*. In conclusion, these results suggest that the intestinal microbiota may play a crucial role in alleviating lactose intolerance, as well as in anti-inflammatory and antioxidant processes.

### 3.3 Pre-supplementary GOS prevented lactose intolerance in mice

#### 3.3.1 Prophylactic supplementation of GOS attenuated the inflammatory responses elicited by lactose intolerance

**FIGURE 3:**
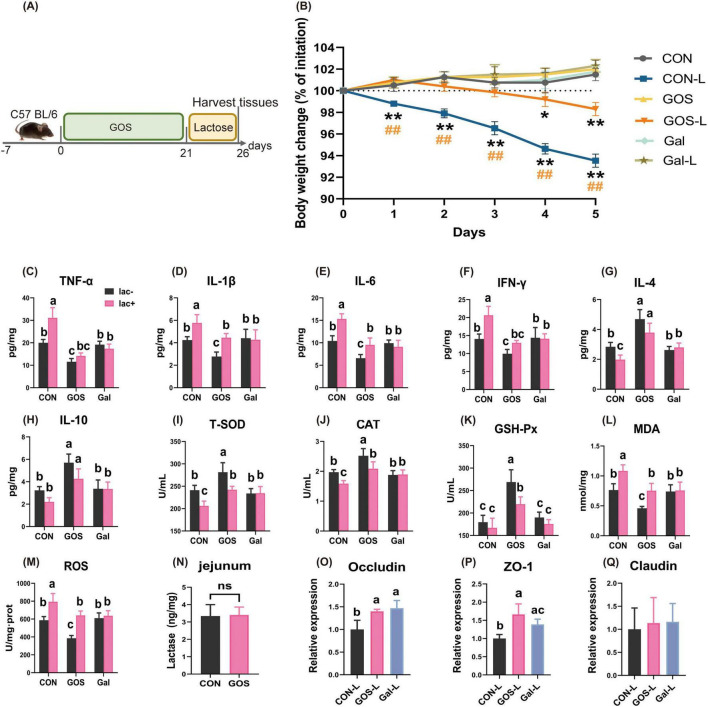
Prophylactic GOS attenuated signs of lactose-induced LI. **(A)** Diagram illustrating the experimental design in this study. **(B)** Daily body weight changes following lactose treatment. Statistical significance was determined using one-way ANOVA, followed by Tukey test. *P ≤ 0.05, **P ≤ 0.01 relative to CON group; ^##^*P* ≤ 0.01 relative to GOS-L group. **(C–H)** Concentrations of four representative pro-inflammatory cytokines, IL-1β, TNF-α, IL-6, IFN-γ, and anti-inflammatory cytokines, IL-4, IL-10 in the ileum. **(I–M)** Prophylactic GOS attenuated oxidative stress and jejunal damage. Concentrations of T-SOD, CAT, GSH-Px, MDA and ROS in the ileum from each group. **(N)** Concentrations of β-galactosidase in jejunum. **(O–Q)** Relative expression jejunal level of Occludin, ZO-1, Claudin mRNA in mice. Data were presented as means ± SEM (*n* = 6 per group). Statistical significance was determined using one-way ANOVA, followed by Tukey test. *P ≤ 0.05, **P ≤ 0.01. ^a-c^Dissimilar letters represent significant difference among different treatments (*P* < 0.05).

Given that GOS is beneficial in reducing risk of colorectal cancer (Piovani et al., 2019), was conducive to intestinal recovery following lactose intolerance, and had diverse effects on the intestinal microbiota, we explored the prophylactic role of GOS against lactose intolerance (Figure 3A).

Prophylactic supplementation with GOS mitigated lactose intolerance in mice, as demonstrated by a significant reduction in daily weight loss assigned to the GOS-L treatment regimen (Figure 3B). Compared with the CON group, GOS significantly decreased contents of IL-1β, IL-6, IFN-γ, and TNF-α, and significantly increased contents of IL-4 and IL-10 in ileal tissue (Figures 3C–H). Galacto-oligosaccharides significantly reduced levels of pro-inflammatory factors in mice ileum and elevated the levels of anti-inflammatory factors; whereas prophylactic supplementation of lactase had no significant impact on the levels of inflammatory factors in the ileum. Thess observations suggest that supplementing GOS reduced the intestinal inflammatory response and enhanced immunity. However, supplementing lactase did not alter the inherent inflammatory response of mice. After administering lactose to mice to induce lactose intolerance, levels of IL-1β, IL-6, IFN-γ, and TNF-α increased significantly compared with CON mice and significantly decreased in the contents of IL-4 and IL-10 in the ileum. In the mice supplemented with lactase, there was no obvious change in the levels of inflammatory factors after continuous administration of lactose, suggesting that lactase effectively degraded lactose without causing lactose intolerance. Nevertheless, after prophylactic supplementation of GOS and subsequent exposure to lactose, levels of IL-1β, IL-6, IFN-γ, and TNF-α were significantly lower than those seen in CON-L mice, and levels of IL-4 and IL-10 were significantly higher. These cytokine concentrations were comparable to the intestinal inflammatory levels in mice supplemented with lactase and then exposed to lactose induction. These results collectively suggest that prophylactic supplementation of GOS significantly suppressed inflammatory responses caused by lactose-induced lactose intolerance, with an effect similar to that of adding lactase.

#### 3.3.2 Prophylactic supplementation of GOS reduced oxidative stress induced by lactose intolerance

Prophylactic supplementation of GOS significantly enhanced the activities of T-SOD, CAT, and GSH-Px (Figures 3I–M), and significantly decreased contents of MDA and ROS in ileum. In contrast, prophylactic supplementation of lactase exerted no significant influence on the oxidative stress level in the ileum of mice. These results suggest that prophylactic supplementation of GOS markedly improved antioxidant capacity of the mouse intestine; but lactase supplementation did not alter the original antioxidant status of mice. In the CON-L group, after administering lactose to mice to induce lactose intolerance, activities of T-SOD, CAT, and GSH-Px decreased significantly, and contents of MDA and ROS increased significantly, indicating that lactose intolerance triggered intense oxidative stress in mice intestine. In the Gal-L group, there was no obvious change in the intestinal oxidative stress level after continuous administration of lactose, suggesting that lactase effectively degraded lactose without causing lactose intolerance. In the GOS-L group, after prophylactic supplementation of GOS and subsequent exposure to lactose challenge, the activities of T-SOD, CAT, and GSH-Px were significantly higher, and the contents of MDA and ROS were significantly lower than those in the CON-L group. In the GOS-L group, antioxidant capacity was slightly higher or comparable to that of mice supplemented with lactase and gavaged with lactose. These results demonstrate that prophylactic supplementation of GOS effectively inhibits intestinal oxidative stress resulting from lactose-induced lactose intolerance. These effects are similar to that of adding lactase.

#### 3.3.3 Prophylactic supplementation of GOS reduced intestinal barrier damage caused by lactose intolerance

Compared with CON, there was no statistically significant difference in the jejunal lactase content of mice with prophylactic supplementation of GOS, indicating that GOS did not affect the expression of intestinal lactase (Figure 3N). The beneficial effects of GOS in alleviating lactose intolerance was not achieved through increasing activity of intestinal lactase. This is consistent with results of Experiment 2.

Prophylactic supplementation of GOS elevated Occludin and ZO-1 in the jejunum in mice (Figures 3O–Q). In the GOS-L group, after receiving prophylactic supplementation of GOS and subsequent lactose induction, mRNA expression levels of Occludin and ZO-1 were significantly higher than those in CON-L mice. Following the lactose challenge, there was no obvious change in levels of mRNA expression for Claudin in jejunum. These results demonstrated that prophylactic supplementation of GOS inhibited damage to the intestinal mucosal barrier caused by lactose intolerance. This response is similar to the response when lactose was supplemented.

#### 3.3.4 Prophylactic supplementation of GOS regulated composition of the intestinal microbiota in lactose-intolerant mice

**FIGURE 4:**
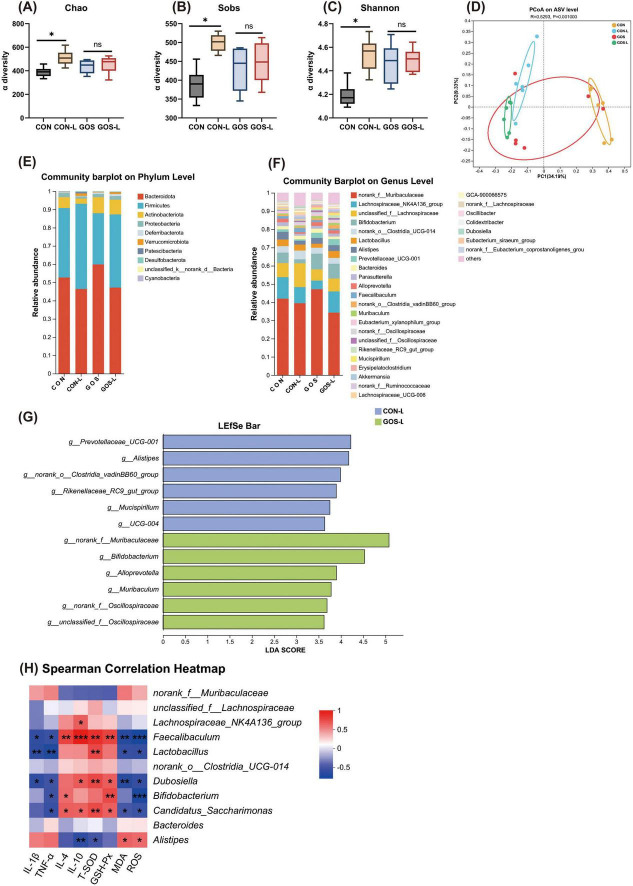
Prophylactic GOS regulated the composition and function of intestinal microbiota. **(A–C)** α-diversity represented by the Chao, Sobs and Shannon index. **(D)** PCoA plots assessed by PERMANOVA among the four groups. **(E,F)** The relative abundance of colonic contents bacterial phyla and genera presented in 99.5% of the community in lactose-treated mice with or without receiving GOS. **(G)** Analysis of differences in the microbial taxa shown by using LEfSe (LDA coupled with effect size measurements). **(H)** Spearman correlation between intestinal microbiota and anti-inflammatory or anti-oxidative parameters among the four groups. The red color denotes a positive correlation, while blue color denotes a negative correlation. The intensity of the color is proportional to the strength of Spearman correlation. Statistical significance was determined using one-way ANOVA, followed by Tukey test. *P ≤ 0.05, **P ≤ 0.01, ***P ≤ 0.001.

The alpha diversity of the cecal microbiota was represented by the Chao index, Sobs index, and Shannon index at the ASV level (Figures 4A–C). After mice consumed lactose for five consecutive days, the Chao, Sobs, and Shannon indexes all increased significantly, indicating that the continuous intake of lactose increased the alpha diversity of the cecal microbiota in mice. Compared with GOS mice, the continuous intake of lactose by mice with prophylactic supplementation of GOS did not significantly alter the Chao, Sobs, and Shannon indexes, suggesting that prophylactic supplementation of GOS prevented the change of alpha diversity of the cecal microbiota in mice when consuming lactose-containing foods. Similarly, including lactose with GOS had no effect on microbial diversity in the cecum compared with CON. PCoA based on the Bray-Curtis distance revealed (Figure 4D) that pre-administration of GOS significantly changed structure of the microbial community in mice. Pre-supplementation of mice with GOS led to a significant separation of the intestinal microbial community structure after lactose intake (*R* = 0.529, *P* = 0.001), indicating that the intestinal microbiota structure of mice was significantly affected after pre-supplementation with GOS and subsequent lactose intake.

Cecal microbiota of mice at the phylum level was still mainly composed of Bacteroidetes and Firmicutes (Figure 4E). In mice pre-supplemented with GOS, influence on composition of the cecal microbiota at the phylum level was manifested as an increased proportion of Actinobacteria when exposed to lactose. At the genus level, prophylactic supplementation of GOS significantly increased *Bifidobacterium* (Figure 4F). *Bifidobacterium* belongs to Actinobacteria, and the increased abundance of *Bifidobacterium* corroborated the results of the increased proportion of Actinobacteria. After receiving a lactose challenge, the proportion of *Bifidobacterium* in mice with prophylactic supplementation of GOS remained almost unchanged, and *Lactobacillus* increased slightly. Whether mice were pre-supplemented with GOS or not, changes in the cecal microbiota upon lactose intake were evident through LEfSe analysis. After lactose intake in CON mice, the proportion of *Alistipes* significantly increased (Figure 4G). After lactose intake with prophylactic supplementation of GOS, *Bifidobacterium* was significantly enriched.

To further understand associations between differential microbiota and inflammatory and antioxidant parameters, a Spearman correlation analysis was performed. The Spearman correlation analysis indicated (Figure 4H) that *Faecalibacterium* was negatively correlated with IL-1β and TNF-α, and positively correlated with IL-4 and IL-10. *Lactobacillus* was positively correlated with T-SOD and GSH-Px, and negatively correlated with MDA and ROS. *Lactobacillus*, *Dubosiella*, *Bifidobacterium*, and *Candidatus Saccharimonas* all exhibited trends very similar to those of *Faecalibacterium*. Which suggests they were all associated with reducing inflammation and oxidative stress in the intestine. These organisms simultaneously seemed to enhance anti-inflammatory and antioxidant capabilities. In contrast, *Alisyipes* was correlated negatively with T-SOD and positively with MDA and ROS. *Alisyipes* seemed to be related to increased levels of oxidative stress and inflammation in the intestine. These results suggest that prophylactic supplementation of GOS regulated composition of the intestinal microbiota. By significantly increasing abundance of probiotics such as *Bifidobacterium*, GOS downregulated levels of inflammatory factors and reduced oxidative stress responses, thereby potentially attenuating the microecological imbalance caused by lactose intolerance.

## 4 Discussion

In humans, LI is usually built up after consuming lactose containing food. There are numerous human reports with varying degree of lactose intolerance (Di Costanzo and Berni Canani, 2018). However, rarely studies using animal model to investigate LI. Here in this study, we developed a mice model by challenging with lactose which demonstrated parallel symptoms of LI as in humans such as higher content of water in stool, bleeding intestines, increased intestinal contents of inflammatory factors and altered intestinal microbiota. The dose of lactose (50 mg/20 g BW) for mice was selected based on the milk content of lactose (25 g/500 mL milk) treated for human (60 kg BW) when measuring LI (Savaiano et al., 2013), and the ratio of surface area vs. BW of mice (Nair and Jacob, 2016). Our results showed that the mice LI model was successful developed.

Experiment 2 confirmed that GOS reduced intestinal damage in mice with lactose intolerance, and alleviated intestinal inflammation and oxidative stress. Compared to healthy individuals, those with acute diarrhea have reduced absorptive capacity, consumed less food, and consequently experienced continuous weight loss (Nyeko et al., 2010). Expression of pro-inflammatory cytokines which such as IL-1β and TNF-α, in the ileum of mice was significantly decreased, downregulated the inflammatory response. We discovered that GOS increased activity of antioxidant enzymes such as T-SOD and GSH-Px, and reduced the concentration of the lipid oxidation end product, MDA, which alleviated cell damage, and mitigated intestinal oxidative stress induced by lactose intolerance. We also found that GOS elevated mRNA expression of the tight junction proteins, Occludin, ZO-1, and Claudin, in the jejunal mucosa which mitigated damage to barrier integrity induced by lactose intolerance. Xi et al. (2023) has demonstrated that GOS improves intestinal barrier function by mediating the NF-κB pathway. All pathological conditions that cause small intestinal damage result in reduced lactase expression (Di Costanzo and Berni Canani, 2018). We considered whether GOS alleviates lactose intolerance in mice based on enhancing small intestinal lactase activity. We observed that addition of GOS after occurrence of lactose intolerance did not significantly alter lactase activity in the jejunum of mice.

We demonstrated that GOS supplementation alter composition of the intestinal microbiota in lactose-intolerant mice. Changes in composition of the intestinal microbiota modify metabolic characteristics and subsequently influence host health (Sharon et al., 2014). PCoA analysis reveals that GOS has significantly influenced the intestinal microbiota structure in lactose-intolerant mice. GOS supplementation significantly increased abundance of *Lactobacillus*. Krumbeck et al. (2018) also observed that GOS promoted colonization of *Lactobacillus* and improved intestinal barrier function in obese individuals. These observations suggest that the upregulation of mRNA expression of intestinal tight junction proteins in lactose-intolerant mice by GOS might be achieved through increasing abundance of *Lactobacillus*.

Endogenous β-galactosidase expressed by intestinal microbiota facilitates utilization of lactose by humans (Gingold-Belfer et al., 2020). *Lactobacillus* produces β-galactosidase to utilize lactose in the intestine and alleviates lactose intolerance (Masood et al., 2011). Spearman correlation analysis demonstrated that relative abundance of *Lactobacillus* was correlated negatively with pro-inflammatory cytokines and positively with antioxidant enzyme activity. A high abundance of *Lactobacillus* was strongly associated with low levels of the pro-inflammatory cytokines, IL-6 and TNF-α. Simultaneously, a high abundance of *Lactobacillus* was strongly associated with a high abundance of the antioxidant enzymes, T-SOD and GSH-Px. *Lactobacillus* secretes lactose permease to exert anti-inflammatory effects by selectively degrading pro-inflammatory chemokines (Von Schillde et al., 2012). In piglets, early supplementation with GOS also enhanced antioxidant enzyme activity and reduced oxidative stress in the intestine (Tian et al., 2022). This range of observations suggest that increase in *Lactobacillus* abundance was related closely to reduction of inflammation and improvement of antioxidant capacity. Conversely, a high abundance of *Alloprevotella* was inseparable from high levels of inflammatory responses and antioxidant stress. *Alloprevotella* is also an important biomarker associated with gastric cancer, as increased abundance is related to a higher risk of gastric cancer (Liu et al., 2023). These results suggest that the alteration of the intestinal microbiota composition caused by GOS played a crucial role in improvement of lactose intolerance, especially the enrichment of *Lactobacillus*.

Supplementation with lactase before consuming doing products can prevent occurrence of clinical symptoms of lactose intolerance. Endogenous lactase functions in the small intestine, especially in the upper part of the small intestine, such as the duodenum and jejunum. Generally, exogenous lactase supplementation mainly functions in the small intestine as well, hydrolyzing lactose into glucose and galactose, and monosaccharides can be directly absorbed by the small intestine. Different lactase products have different optimal pH ranges. Exogenous lactase supplementation no longer functions in the large intestine because the intestinal microecology environment of the large intestine, such as pH, is not suitable for the activity of lactase (Ibba et al., 2014; Ojetti et al., 2010). To assess the prophylactic effect of GOS on lactose intolerance, a positive control group, Gal-L, was established, where mice were given lactase 30 min before the lactose gavage. Mice in this group did not exhibit signs of lactose intolerance. To eliminate other influences of lactase addition on mice, a 21-day experiment with lactase addition was also conducted.

Similar to therapeutic administration of GOS, prophylactic supplementation of GOS was equally effective in alleviating inflammation in the ileum of lactose-intolerant mice, as manifested by decreased levels of pro-inflammatory cytokines and increased levels of anti-inflammatory cytokines. This response was comparable to the levels of inflammatory factors observed in the Gal-L group which indicates that prophylactic supplementation of GOS can achieve the same effect as adding lactase in reducing intestinal inflammation caused by lactose intolerance. Furthermore, prophylactic administration of GOS increased the activities of antioxidant enzymes such as CAT, T-SOD, and GSH-Px in the ileum, and reduced production of harmful substances associated with oxidative stress, such as MDA and ROS. Collectively, these responses indicated that GOS significantly enhanced the antioxidant status of the intestine, effectively reduced oxidative stress in mice ileum, and the retention of antioxidant enzyme activity could still reach the level of the Gal-L group, suggesting that prophylactic supplementation of GOS enhanced the intestinal antioxidant capacity and achieved the same effect as adding lactase. Prophylactic supplementation of GOS did not increase the activity of lactase in the jejunal mucosa. Similar to results reported by Leforestier et al. (2009). After feeding mice GOS for 4 weeks, activity of lactase did not undergo significant changes (Leforestier et al., 2009), indicating that the beneficial effect of GOS in preventing lactose intolerance was not achieved by increasing the activity of jejunal lactase. Prophylactic supplementation of GOS increased mRNA expression of tight junction proteins in jejunal mucosa, and mRNA expression of Occludin, ZO-1, and Claudin decreased slightly after lactose intake. Prophylactic supplementation of GOS protected integrity and function of the intestinal barrier after lactose intake, enhanced intestinal tolerance to lactose, and reduced intestinal damage caused by lactose intolerance.

After prophylactic supplementation of GOS, cecal microbial composition of individual mice with lactose intolerance underwent significant changes. The most notable change was the increased the abundance of lactose-metabolizing bacteria. These changes were similar to alterations in the fecal microbiome of lactose-intolerant subjects participating (Azcarate-Peril et al., 2017). In mice receiving prophylactic supplementation of GOS, there was no significant change in community diversity at the ASV level, but results of PCoA analysis indicated a significant alteration in the microbial community structure. Prophylactic supplementation of GOS significantly increased abundance of *Bifidobacterium* and decreased abundance of *Alistipes*. In mice that received prophylactic supplementation of GOS and ingested lactose, the abundance of *Bifidobacterium* did not change significantly, while the abundance of *Lactobacillus* showed a marked increase. These indicated that the changed microbiota played a crucial role in alleviating the development of lactose intolerance. Almeida et al. (2012) found that a 4-week supplementation of *Lactobacillus* and *Bifidobacterium* preparations in lactose-intolerant patients achieved the same effect as supplementing with lactase, and that effect lasted for up to three months. However, there remains no clear conclusions regarding the effective dose and duration of probiotic supplementation. Therefore, more research is needed to evaluate the optimal dose and most suitable supplementation time of combined probiotics to obtain optimal results in improving lactose intolerance. *Bifidobacterium* can metabolize oligosaccharides, inhibit inflammatory responses, and regulate host immunity (Kitaoka, 2012; Chen et al., 2021). *Bifidobacterium* significantly enriched phospho-β-galactosidase and the metabolic pathways for digesting lactose (Caspi et al., 2020). Additional benefits of *Bifidobacterium* as a probiotic on the intestinal tract include protecting the intestinal tract from inflammatory diseases and colorectal cancer, mainly through inhibiting the NF-κB signaling pathway and inflammatory responses (Huang et al., 2017; Celiberto et al., 2017; Paveljšek et al., 2018). Activation of NF-κB is an important pro-inflammatory cellular mediator involved in the occurrence of colon cancer (Han et al., 2016; Huang et al., 2017). *Faecalibacterium* is regarded as an anti-inflammatory genus, because its metabolites can block NF-κB activation. Spearman correlation analysis revealed positive correlations between the abundances of *Bifidobacterium*, *Lactobacillus*, and *Faecalibacterium* with antioxidant capacity and the ability to inhibit inflammatory responses in the intestinal tract of mice. *Dubosiella* enhances immune tolerance of the body (Zhang et al., 2024). *Candidatus Saccharimonas* protects intestinal structure, increases intestinal antioxidant enzymes and activity, and consequently reduces intestinal inflammatory diseases (Cruz et al., 2020). Prophylactic supplementation of GOS decreased abundance of *Alistipes*. *Alistipes* has pathogenic effects on intestinal inflammatory diseases, colorectal cancer, and was associated with mental symptoms of depression (Moschen et al., 2016). Reducing the abundance of *Alistipes* may be associated with a decreased risk of intestinal diseases. In conclusion, the protective effect of prophylactic supplementation of GOS is highly correlated with a significant increase in abundance of *Bifidobacterium*, which possesses anti-inflammatory and anti-tumor properties. Changes in the composition of the intestinal microbiota contribute significantly to enhancing intestinal tolerance to lactose. A recent study by Wu et al. (2024) revealed that GOS enriched *Lactobacillus reuteri* and secondarily promoted with the production of pentadecanoic acid to alleviate intestinal dysfunction. Our results found similar enrichment of *Lactobacillus*. We also measured intestinal pH values which showed slightly lower pH in LI mice than control (data not shown). These findings were consistently with the reported study of Wu et al. (2024).

Our research results indicated that GOS mediated the microbial community. Particularly, the enrichment of *Lactobacillus* and *Bifidobacterium* played a crucial role in alleviating lactose intolerance and enhancing intestinal tolerance to lactose. The increase in microbes mediated by GOS might also be involved in maintaining homeostatic balance in the intestinal tract by improving colonic epithelial integrity and mucosal immunity (Kayama and Takeda, 2020). Eventually, homeostasis of intestinal epithelial cells is strengthened, and tolerance to lactose is concurrently enhanced which reduces the occurrence of lactose intolerance.

In summary, supplementing GOS to mice mitigated their signs of lactose intolerance and favorably altered composition of the intestinal microbiota in mice. Conversely, when lactose-intolerant mice were pre-supplemented with GOS and then exposed to lactose, similar changes occurred in composition of the intestinal microbiota in mice, with an increase in the abundance of probiotics. Spearman correlation analysis implied that enrichment of probiotics following alteration of the intestinal microbiota mediates beneficial effects of anti-inflammation, antioxidation, and maintaining intestinal integrity, thereby achieving the effect of preventing lactose intolerance.

## 5 Conclusion

We discovered that GOS reduced inflammatory response, enhanced antioxidant capacity, and regulated the composition of the intestinal microbiota, and ultimately alleviated the signs of lactose intolerance. Enriched probiotics such as *Lactobacillus* and *Bifidobacterium* showed a negative correlation with the pro-inflammatory factor TNF-α, promoting intestinal anti-inflammatory and increasing intestinal tolerance to lactose. These findings offer novel insights into using GOS as an alternative to lactase for preventing lactose intolerance and will facilitate development of prevention strategies for lactose intolerance and other intestinal disorders.

## Data Availability

The data presented in the study are deposited in the NCBI repository, accession number: PRJNA1209674, PRJNA1209660.

## Footnotes

1
https://cloud.majorbio.com

2
http://www.mothur.org/wiki/calculators
