# Supplementary material for: Galacto-oligosaccharides alleviate experimental lactose intolerance associated with gut microbiota in mice
Source: Front Microbiol. 2025 Mar 25;16:1530156. doi: 10.3389/fmicb.2025.1530156 (PMC11975899; doi:10.3389/fmicb.2025.1530156)
Supplement: Supplementary file 1 [file Data_Sheet_1.pdf]

AIN-93G Growth Purified Diet (also known as #5801-G) 57W5

DESCRIPTION

TestDiet® AIN-93G Growth Purified Diet is the growth diet for rodents recommended by the American Institute of Nutrition. It is formulated to substitute for the previous version (AIN-76A) to improve animal performance.

Storage conditions are particularly critical to TestDiet® products, due to the absence of antioxidants or preservative agents. To provide maximum protection against possible changes during storage, store in a dry, cool location. Storage under refrigeration (2° C) is recommended. If long term studies are involved, store the diet at -20° C or colder. Be certain to keep in air tight containers.

*Product Forms Available**	*Catalog #*
1/2" Pellet	7597
1/2" Pellet, Irradiated	1810393
Meal	1810538
Meal, Irradiated	1810539

**Other Forms Available By Request*

TYPICAL ANALYSIS

Protein	18.7%
Fat	7.0%
Fiber	5.0%
Carbohydrate	64.7%
Metabolizable Energy	3.97 kcal/gm

INGREDIENTS (%)

Corn Starch	39.7486
Casein - Vitamin Free	20.0000
Maltodextrin	13.2000
Sucrose	10.0000
Soybean Oil	7.0000
Powdered Cellulose	5.0000
AIN 93G Mineral Mix	3.5000
AIN 93 Vitamin Mix	1.0000
L-Cystine	0.3000
Choline Bitartrate	0.2500
t-Butylhydroquinone	0.0014

FEEDING DIRECTIONS

Feed ad libitum to mice and rats. Plenty of fresh, clean water should be available at all times.

CAUTION:
Perishable, upon receipt store in a cool dry place, refrigeration recommended.

For laboratory animal experimental use only, NOT for human consumption.

8/12/2010

NUTRITIONAL PROFILE [1]

Protein, %	***18.3***
Arginine, %	0.70
Histidine, %	0.52
Isoleucine, %	0.96
Leucine, %	1.73
Lysine, %	1.45
Methionine, %	0.52
Cystine, %	0.37
Phenylalanine, %	0.96
Tyrosine, %	1.01
Threonine, %	0.77
Tryptophan, %	0.22
Valine, %	1.14
Alanine, %	0.55
Aspartic Acid, %	1.29
Glutamic Acid, %	4.08
Glycine, %	0.39
Proline, %	2.36
Serine, %	1.10
Taurine, %	0.00
Fat, %	***7.1***
Cholesterol, ppm	0
Linoleic Acid, %	3.58
Linolenic Acid, %	0.55
Arachidonic Acid, %	0.00
Omega-3 Fatty Acids, %	0.55
Total Saturated Fatty Acids, %	1.05
Total Monounsaturated Fatty Acids, %	1.54
Polyunsaturated Fatty Acids, %	3.78
Fiber (max), %	***5.0***
Carbohydrates, %	***63.2***
Energy (kcal/g) [2]	***3.89***

From:	kcal	%
Protein	0.731	18.8
Fat (ether extract)	0.637	16.4
Carbohydrates	2.528	65.1

Minerals

Calcium, %	0.51
Phosphorus, %	0.32
Potassium, %	0.36
Magnesium, %	0.05
Sodium, %	0.13
Chlorine, %	0.22
Fluorine, ppm	1.0
Iron, ppm	39
Zinc, ppm	35
Manganese, ppm	11
Copper, ppm	6.0
Cobalt, ppm	0.0
Iodine, ppm	0.21
Chromium, ppm	1.0
Molybdenum, ppm	0.14
Selenium, ppm	0.24

Vitamins

Vitamin A, IU/g	4.0
Vitamin D-3 (added), IU/g	1.0
Vitamin E, IU/kg	81.6
Vitamin K (as menadione), ppm	0.29
Thiamin Hydrochloride, ppm	6.1
Riboflavin, ppm	6.7
Niacin, ppm	30
Pantothenic Acid, ppm	16
Folic Acid, ppm	2.1
Pyridoxine, ppm	5.8
Biotin, ppm	0.2
Vitamin B-12, mcg/kg	29
Choline Chloride, ppm	1,250
Ascorbic Acid, ppm	0.0

1. Formulation based on calculated values from the latest ingredient analysis information. Since nutrient composition of natural ingredients varies and some nutrient loss will occur due to manufacturing processes, analysis will differ accordingly. Nutrients expressed as percent of ration on an As Fed basis except where otherwise indicated.
2. Energy (kcal/gm) - Sum of decimal fractions of protein, fat and carbohydrate x 4,9,4 kcal/gm respectively.

ISO 9001:2000 CERTIFIED

TestDiet

www.testdiet.com
