# Supplementary material for: Galacto-oligosaccharides alleviate experimental lactose intolerance associated with gut microbiota in mice
Source: Front Microbiol. 2025 Mar 25;16:1530156. doi: 10.3389/fmicb.2025.1530156 (PMC11975899; doi:10.3389/fmicb.2025.1530156)
Supplement: Supplementary file 2 [file Data_Sheet_2.pdf]

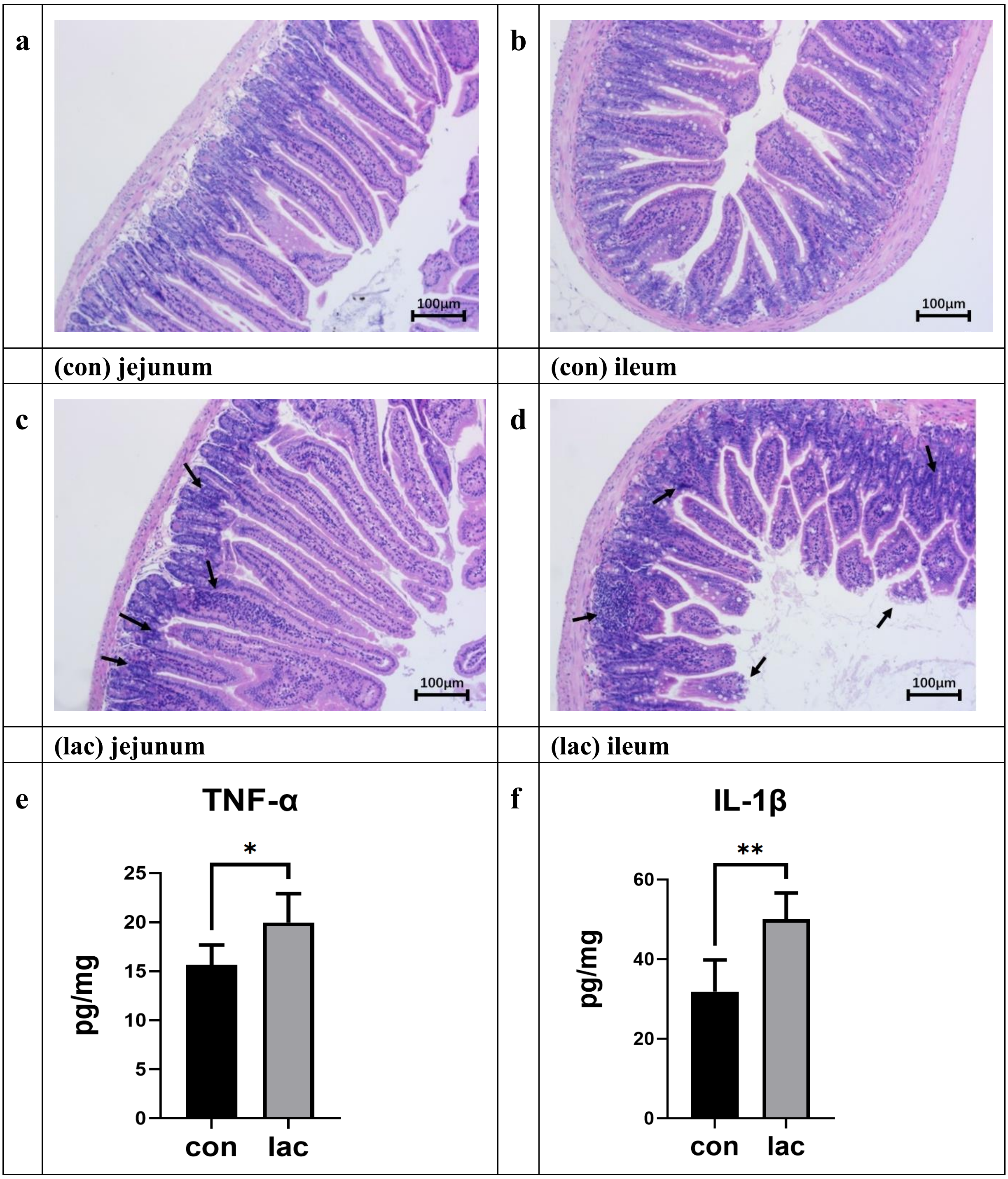
a
b
100µm
100µm
(con) jejunum
(con) ileum
c
d
100µm
100µm
(lac) jejunum
(lac) ileum
e
TNF-α
*
pg/mg
25
20
15
10
5
0
con
lac
f
IL-1β
**
pg/mg
60
40
20
0
con
lac

g

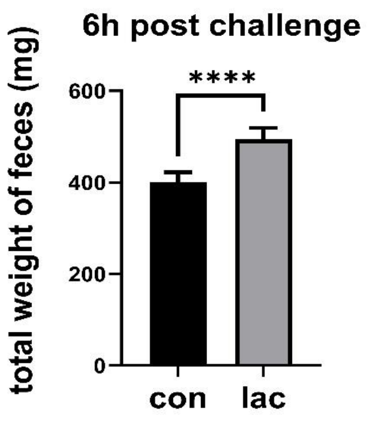

h

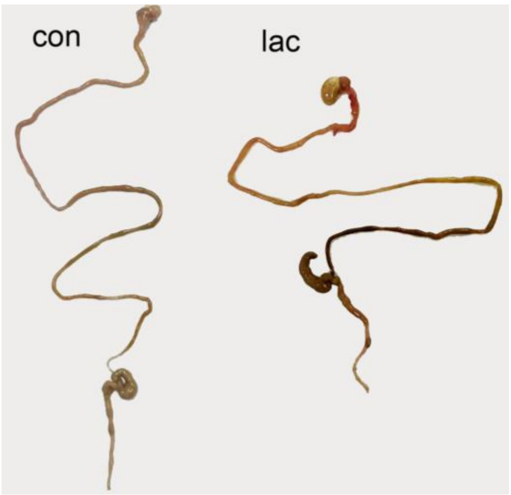

Figure S. Lactose-induced experimental LI.

a-d, H&E staining of the jejunum and ileum in mice with or without lactose challenged. **e-f,** Concentrations of two representative pro-inflammatory cytokines, TNF-α and IL-1β in the ileum. **g,** Total weight of feces following lactose challenge. **h,** LI mice morphology on day 7 of lactose challenge. Data were presented as means ± SEM (n = 8 per group). Statistical significance was determined using one-way ANOVA, followed by Tukey test. *P ≤ 0.05, **P ≤ 0.01.
